# The cardio‐renal‐metabolic role of the nod‐like receptor protein‐3 and senescence‐associated secretory phenotype in early sodium/glucose cotransporter‐2 inhibitor therapy in people with diabetes who have had a myocardial infarction

**DOI:** 10.1111/dme.70059

**Published:** 2025-04-25

**Authors:** M. U. Shah, C. L. Cliff, P. E. Squires, K. Lee, C. E. Hills

**Affiliations:** ^1^ Cardiorenal Group, Diabetes, Metabolism, & Inflammation Joseph Bank Laboratories, University of Lincoln Lincoln UK; ^2^ Lincoln Heart Centre United Lincolnshire Hospitals Lincoln UK

**Keywords:** acute myocardial infarction (AMI), inflammation, macrophages, NOD‐like receptor protein 3 (NLRP3), senescence, sodium/glucose cotransporter‐2 inhibitors (SGLT2i), type 2 diabetes mellitus (T2DM)

## Abstract

**Aims:**

Following an acute myocardial infarction (AMI), individuals with type 2 diabetes (T2DM) have a 2‐to‐3 fold increased risk of mortality compared to those without diabetes, and globally cardiorenal complications account for 50% of diabetes‐related deaths. The use of sodium/glucose cotransporter‐2 inhibitors (SGLT2i) in people with T2DM‐AMI is associated with decreased inflammatory burden and improved cardiorenal outcomes. The mechanisms behind this protection are unclear and form the basis of this study.

**Methods:**

This single centre, prospective study with randomisation will utilise plasma and monocyte‐derived macrophages from patients with T2DM who have recently had an AMI and are prescribed Empagliflozin (SGLT2i) either immediately following the acute cardiac event or at 3 months post‐AMI.

**Results:**

The study will test the hypothesis that Empagliflozin provides anti‐inflammatory protection by suppressing systemic NOD‐like receptor protein‐3 (NLRP3) inflammasome activation and the pro‐inflammatory senescence‐associated secretory phenotype (SASP), perpetrators of sterile (non‐pathogen evoked) inflammation linked to poor clinical outcomes in T2DM‐AMI patients. The study will also assess the benefits of early intervention on these parameters.

**Conclusions:**

Elucidating a role for an SGLT2i in suppressing sterile inflammation will enhance understanding of how they can be used effectively to treat cardiorenal complications and will identify novel pathways for future intervention. Furthermore, the optimal timing of when to initiate SGLT2i therapy post‐AMI is unclear. Correlating the level of protection to the onset of therapy in individuals with T2DM, AMI and at cardiovascular risk will establish if Empagliflozin provides greater benefit when intervention is initiated earlier.

## 1 INTRODUCTION

Type 2 diabetes mellitus (T2DM) is a major risk factor for premature onset of multiple age‐related comorbidities and complications, including cardiovascular and kidney disease.^1^ Acute myocardial infarction (AMI) is the primary cause of death in individuals with T2DM, where patients with diabetes have at least a twofold increased risk of mortality compared to those without the disease, both during and substantially after the acute event.^2^ Following an AMI, patients are at a higher risk of developing kidney complications whilst those with pre‐existing kidney injury are more likely to suffer a subsequent cardiovascular event, a link referred to as the ‘cardiorenal syndrome’. The reciprocal relationship between cardiovascular and kidney injury develops because of multi‐organ crosstalk and accounts for more than half of deaths in those with diabetes. Prescribed originally to regulate blood glucose in people with T2DM, the sodium/glucose cotransporter‐2 inhibitors (SGLT2i's) have since demonstrated improved cardiorenal outcomes in people with^3^, ^4^ and without diabetes.^5^, ^6^ However, despite the wealth of evidence illustrating multi‐organ benefits, there remains a paucity of mechanistic data explaining how these compounds elicit their cardiorenal effects. Understanding fundamental processes for how SGLT2i's protect in vivo, is critical in explaining the clinical benefits seen across the heart and kidneys.

To date, studies investigating the cellular mechanisms of SGLT2i's have uncovered a role for Empagliflozin in inhibiting NOD‐like receptor protein‐3 (NLRP3) inflammasome activation when prescribed to patients with T2DM at high cardiovascular risk for 30 days.^7^ Activation of the NLRP3 inflammasome and our innate immune response is a major perpetrator of inflammatory damage in both AMI^8^ and chronic kidney disease (CKD),^9^ with NLRP3 activation linked to renal injury‐induced cardiac dysfunction^10^ and the accumulation of senescent cells.^11^ Senescent cells produce a pro‐inflammatory senescence‐associated secretory phenotype (SASP) which exacerbates downstream sterile inflammation. Under sterile conditions, NLRP3 is activated by danger‐associated molecular patterns (DAMPs) including adenosine triphosphate (ATP). These DAMPs are released into the intercellular space from connexin hemichannels, pores in the cell membrane which open in response to injury. Linked to the underlying pathology of chronic conditions of inflammation, aberrant connexin hemichannel activity is a recognised therapeutic target.^12^, ^13^, ^14^

Examining the cardio‐metabolic role of NLRP3 and SASP in early SGLT2i therapy is a single centre, prospective study with randomisation that will determine if early treatment with SGLT2i (Empagliflozin) is beneficial over delayed intervention in reducing sterile inflammation in patients with T2DM who have recently had an AMI. Whilst SGLT2i's are approved for use in patients with T2DM following an AMI, current prescription patterns show a delay between the cardiac event and SGLT2i prescription. Through assessment of (i) NLRP3 inflammasome activation; (ii) aberrant Cx43 hemichannel‐mediated ATP release and (iii) the senescence‐induced SASP, we hypothesise that early Empagliflozin therapy immediately following an AMI blunts inflammation and reduces cell senescence in comparison to delayed therapy at 3 months' time. Understanding the effect and timing of SGLT2i prescription on these events will enable timely intervention to help reduce residual inflammatory risk following an AMI and optimise therapeutic application in a high‐risk cohort.

## 2 MATERIALS AND METHODS

### 2.1 Study design

This is a single centre, prospective study with randomisation, involving working with human blood samples.

### 2.2 Study outcome

The primary objective is to assess whether Empagliflozin commenced immediately after an AMI blunts priming/activation of the NLRP3 inflammasome, suppresses Cx43 hemichannel‐mediated ATP release and reduces senescent cell accumulation and its SASP. We also aim to assess whether the beneficial effects of Empagliflozin are greater when prescribed immediately, compared to when therapy is initiated at 3 months post‐AMI.

### 2.3 Study population

#### 2.3.1 Recruitment

We aim to recruit 66 participants (30 for each Arm [early versus delayed] with 10% over‐recruitment as contingency for drop‐out) from the in‐patient cardiology wards at Lincoln County Hospital (a teaching district general hospital, United Kingdom). The rationale for selecting 30 patients per cohort is based on successful outcomes reported in a study by Kim et al where the authors established a role for Empagliflozin in blocking the NLRP3 inflammasome in 29 patients with T2DM at risk of CVD when prescribed SGLT2i for 30days.^7^ Eligible individuals comprised those with T2DM and admitted with AMI who are not already on an SGLT2i, who will be identified by the members of the direct clinical care team following a review of the medical notes and blood results.

#### 2.3.2 Eligibility criteria

Inclusion criteria:

- Male and female patients aged 18 to 84 years
- Patients with known or new T2DM and newly diagnosed AMI
- Eligible for SGLT2i therapy AND not currently prescribed an SGLT2i
- The patient is eligible for both prescribing pathways for starting SGLT2i:
  ◦ Starting SGLT2i prior to discharge or
  ◦ Starting SGLT2i at follow‐up clinic
- The patient has no preference for a specific prescribing pathway and consents to be randomised.
- The patient can provide informed consent

Exclusion criteria:

- Pregnancy or breast feeding
- Severe end‐stage kidney disease
- Severe end‐stage liver disease
- Other conditions that would reduce the expected life span of a patient to less than 2 years
- Unable to provide informed consent
- Patients who have an indication for early start of or are already prescribed, Empagliflozin/ other SGLT2i, separate from the above conditions (e.g., patients with known symptomatic heart failure with reduced ejection fraction [EF <40%] or already prescribed SGLT2i for known T2DM)
- Acute renal failure
- Cardiogenic shock
- Severe valvular heart disease
- Surgical revascularisation
- Inflammatory‐related conditions, including infection, cancer or autoimmune disease
- Female participants will be assessed to determine whether they are of childbearing potential. Non‐childbearing potential is defined as women who are either permanently sterilised (bilateral salpingectomy/oophorectomy or hysterectomy) or if they are post‐menopausal, i.e., amenorrhoeic (cessation of monthly period), for at least 12 months following cessation of all exogenous hormone therapy and either:
  ◦ Aged older than 50 years, or
  ◦ Aged 50 years or less and have follicle‐stimulating hormone levels in the postmenopausal range
  ◦ Participants who do not fulfil these criteria will be offered a urine pregnancy test, as per clinical assessment, to rule out pregnancy

Female participants deemed of child bearing age who are keen to participate and not pregnant at the time of recruitment will be counselled on the potential effects of Empagliflozin on pregnancy at the time of randomisation along with advice on contraception for the duration of the study if required. Adherence will be checked, as and where appropriate, for the duration of the study.

### 2.4 Study visits and techniques employed

**FIGURE 1:**
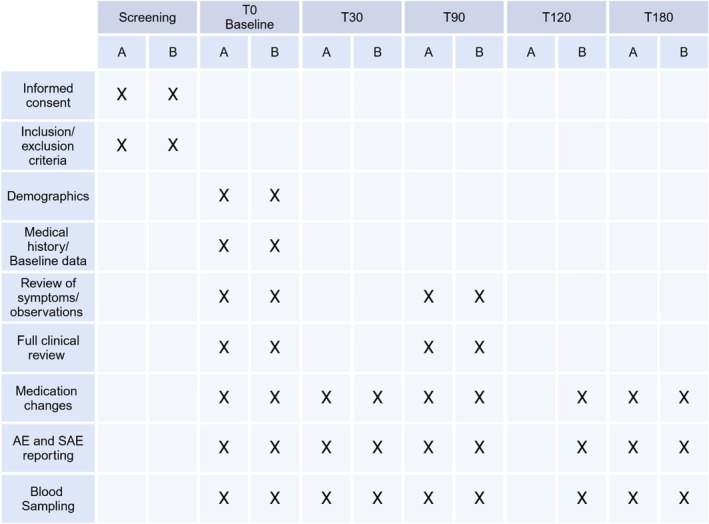
Outline of visits during follow up for participants randomised to one of 2 groups (A or B). AE, adverse event; SAE, serious adverse events. (Figure generated using Biorender software.)

**FIGURE 2:**
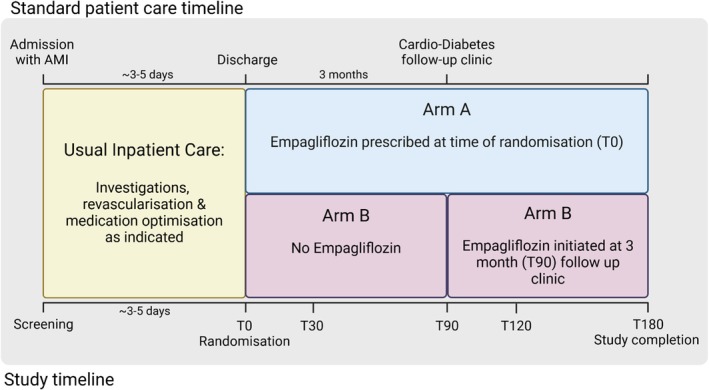
Schematic showing two arms of the study and timing of initiation of Empagliflozin. AMI, acute myocardial infarction. (Figure generated using Biorender software.)

**FIGURE 3:**
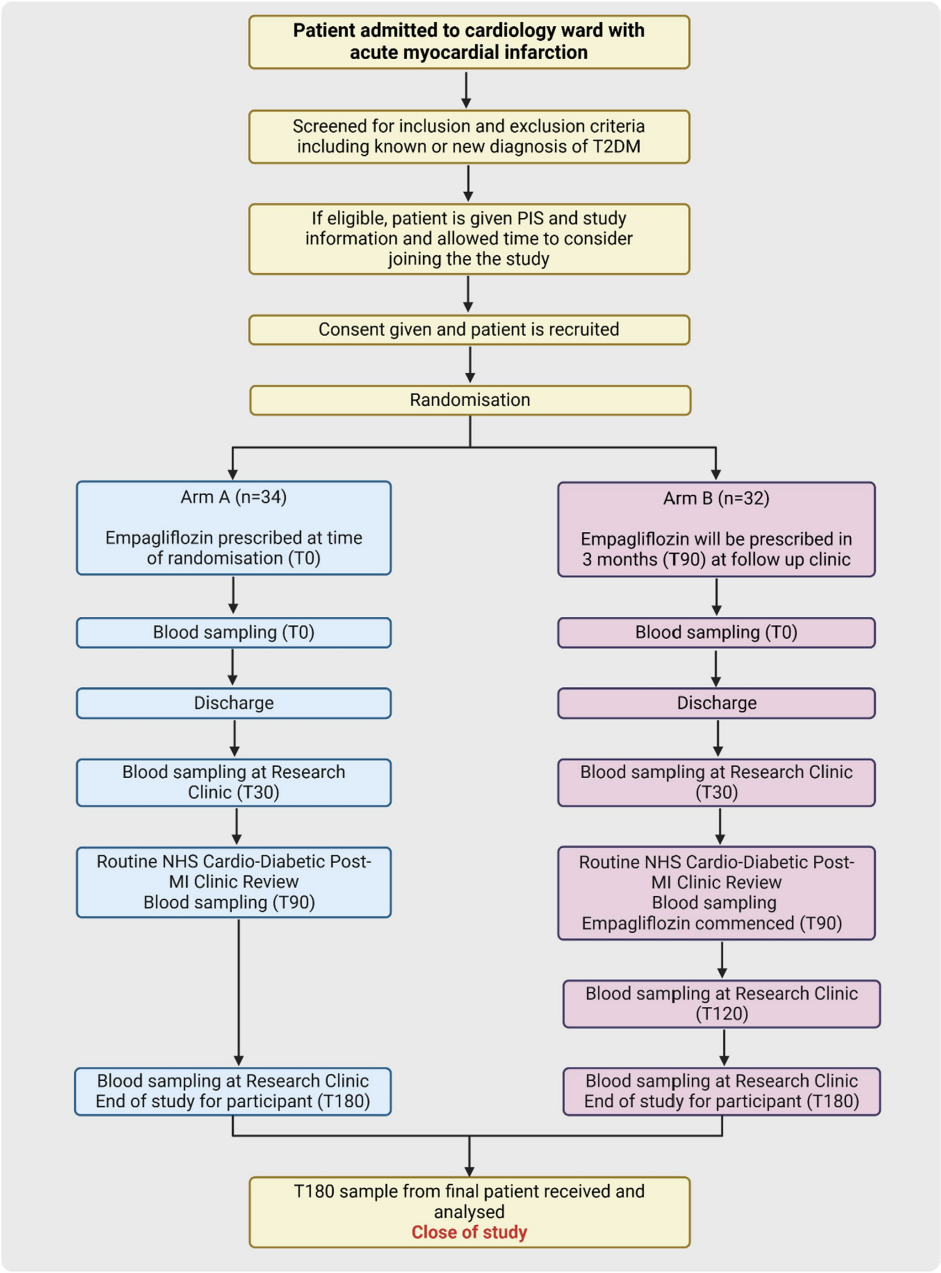
Flowchart detailing participant journey in the study and timing of initiation of Empagliflozin. MI, myocardial infarction; NHS: National Health Service; PIS, patient information sheet, T2DM, type 2 diabetes mellitus. (Figure generated using Biorender software.)

A summary of study design is presented in Figures 1, 2, 3.

#### 2.4.1 Randomisation and intervention

Individuals will be randomly allocated, using unmarked envelopes, to either early (immediate) or delayed (3 months) Empagliflozin 10 mg, once daily, in a 1:1 ratio. At the time of recruitment and randomisation, baseline characteristics will be recorded as per the baseline data collection form (Supplementary).

Patients in both Arms will be followed for 6 months with 3 follow‐up visits for Arm A (days 30, 90 and 180) and 4 visits for Arm B (days 30, 90, 120 and 180) participants. Samples for biomolecular analysis will be obtained at each visit, with additional blood tests for clinical assessment performed at days 90 and 180.

#### 2.4.2 Blood sampling

Blood samples, approximately 35–40 mL, will be obtained prior to discharge and then at regular intervals in the study. The blood samples will be transferred, via an approved cold chain process, to the University of Lincoln (UoL) research laboratories for further analysis. Evidence for successful implementation of in vitro assays is based on previously published and preliminary data from our group.^12^, ^15^

#### 2.4.3 Sample analysis

We will employ established/validated assays to quantify markers of NLRP3 inflammasome activation, cell senescence, its SASP and Cx43 hemichannel activity in plasma and monocyte‐derived macrophages (MDMs) from blood collected from patients. Peripheral blood mononuclear cells (PBMCs) will be isolated using density gradient centrifugation, from which monocytes will be isolated by CD14+ magnetic bead isolation (Miltenyi Biotec, Germany). Monocytes will be seeded and treated with macrophage colony‐stimulating factor (MCSF) for 6 days, with media replaced on day 3 to induce differentiation to monocyte‐derived macrophages (MDMs). Plasma will be collected during PBMC isolation and stored at −80°C. Experiments will be carried out in triplicate.

Analysis will be carried out within each Arm and across multiple time points. We will study, (i) the effect of Empagliflozin versus no drug on signalling pathways/markers of interest (Arm A and B 0‐to‐90 days), (ii) the longevity of benefit (Arm A 0‐to‐180 days) and (iii) if these benefits are greater when therapy commences early (Arm A 0‐to‐90 days) compared to 90 days post‐acute event (Arm B 90‐to‐180 days).

#### 2.4.4 Caspase Glo‐1 inflammasome assay

The Caspase‐Glo® 1 Inflammasome Assay (Promega, US) is a simple, homogeneous, bioluminescent method to selectively measure the activity of caspase‐1, a gold standard measure of inflammasome activation. Caspase‐1 will be measured in patient MDMs stimulated with a combination of lipopolysaccharide (LPS) and ATP. A caspase inhibitor (YVAD CMK) will confirm caspase‐1 specificity.

#### 2.4.5 Quantitative real‐time PCR

We will determine if established pro‐inflammatory markers (interleukin‐1 beta (IL1β), tumour necrosis factor alpha (TNF‐α) and interleukin‐6 (IL6)) and markers of cell senescence (p21, p27, B‐cell leukemia/lymphoma 2 protein (BCL2), BCL2‐associated X (BAX), interleukin‐8 (IL8) and interleukin‐11 (IL11)) are decreased in unstimulated patient MDMs. Total RNA will be extracted using TRIzol reagent (Invitrogen, US), chloroform and isopropanol and converted to complementary (c)DNA using a high‐capacity cDNA reverse transcription kit (Applied biosystems, US) following the manufacturer's instructions. Quantitative real‐time (qRT) polymerase chain reaction (PCR) will be performed using qPCRBIO SyGreen Blue Mix, ROX dye (PCR Biosystems, UK) and DNA oligo primers to detect altered mRNA expression of key markers of interest.

#### 2.4.6 ATP release

Patient MDMs will be treated with the anti‐ectonucleotidase ARL 67156 trisodium salt ± hemichannel blockers Tonabersat or Peptide 5 for 2 h, followed by LPS stimulation. Extracellular ATP will be measured using the ATP‐lite luminescence assay system (Perkin Elmer, US) following the manufacturer's instructions.

#### 2.4.7 Inflammatory marker release

Lumit immunoassays (Promega, Madison, US) will determine changes in the secretion of inflammatory and SASP markers IL1β, IL6 and TNFα in LPS (IL6 and TNFα) or LPS + ATP (IL1β) stimulated cells.

#### 2.4.8 Plasma biomarker detection

The ELLA™ automated immunoassay (Bio‐Techne) will measure key SASP proteins monocyte chemoattractant protein‐1 (MCP‐1), matrix metalloproteinase‐9 (MMP‐9), osteopontin (OPN) and Serpin E1 in patient plasma samples.

### 2.5 Safety

Empagliflozin is licensed for use in the treatment of patients with T2DM, cardiovascular disease and AMI. Potential participants will be assessed for important contraindications, including diabetic ketoacidosis, active foot ulceration and CKD stage 5. Eligible patients will be included in the study and commenced on guideline‐directed medical therapy. Therefore, no investigational medication will be introduced, and patients will be commenced, if appropriate, on already established drugs licensed for use in these presentations. Any adverse events (AEs) or serious adverse events (SAEs) identified will be managed as part of the usual standard of care and recorded for the purpose of the study.

### 2.6 Data collection and analysis

Pseudo‐anonymised coded patient data, demographics, clinical data and specific clinical results and measurements of interest and relevance will be collected for final data analysis. All data will be documented in English. Data will be stored in electronic format using Microsoft Access and statistical analysis performed using GraphPad Prism, Strata and R. Expression data and functional measurements will mostly be contained in simple small file size spreadsheets and will be made available in supplementary material accompanying papers under Creative Commons Attribution‐Non‐commercial‐NoDerivs (CC BY‐NC‐ND) gold access for immediate and permanent read and download. In addition, these data will be hosted on secure servers on the Lincoln Repository.

A normality test will be performed for all continuous variables. Data will be presented as means with standard error of the mean or medians with interquartile range. Intergroup comparisons of laboratory results and outcome measures will be made by Welch's *t* test or a repeated measures one‐way ANOVA with Tukey's mixed effects analysis, where *p* < 0.05 will be considered statistically significant.

### 2.7 Withdrawal

Participants may be withdrawn from the trial either at their own request or at the discretion of the investigator. The participants will be made aware that this will not affect their future care.

### 2.8 Study management

The study is registered ISRCTN12589919, and the protocol is approved by East of Scotland Research Ethics Service (22/ES/0047) and Health Research Authority (319343) in December 2022.

## 3 DISCUSSION

SGLT2i's are a class of glucose‐lowering medications with established cardio‐reno‐vascular benefits in individuals with T2DM and at high risk of further cardiovascular events. However, the standard of care in UK practice conservatively follows known randomised clinical trial (RCT) protocol‐based practice (as patients with recent acute events such as AMI were usually excluded in RCTs), thus common clinical practice delays initiation (up to 3–6 months) after an AMI event. Compelling RCT evidence suggests benefit occurs early in the initiation of SGLT2i therapy and persists.^16^ Furthermore, recent findings reported from a real‐world observational study from the Swedish Web‐system for Enhancement and Development of Evidence‐based care in Heart disease Evaluated According to Recommended Therapies (SWEDEHEART) registry suggest a 30% reduction in cardiovascular outcomes of hospitalisation for heart failure and death when initiated after an AMI in those with T2DM.^17^ Combined with good safety profiles in patients with previous and recent AMI, this recommends that early intervention may confer greater clinical benefit. This study aims to identify the optimal time of SGLT2i intervention to give the greatest benefit to patients with T2DM and an AMI, to better inform prescription patterns.

Like other chronic age‐related diseases, T2DM and its complications are caused by the convergence of fundamental mechanisms that underlie age‐related tissue dysfunction, including chronic ‘sterile’ NLRP3‐induced inflammation^18^ and cellular senescence.^19^, ^20^ In vivo studies have reported a role for SGLT2i's in suppression of NLRP3^21^, ^22^, ^23^ and senescence,^24^ potential mechanisms mediating their protective cardiovascular effects. Additionally, a recent study in Nature Communications reported that 30‐day treatment with SGLT2i Empagliflozin reduced NLRP3 activity (decreased IL1β and TNFα levels) in MDMs from patients with T2DM and at high‐risk of CVD.^7^ Despite these exciting observations, we do not know if SGLT2i's can decrease levels of these inflammatory proteins in patients with T2DM when prescribed at different intervals after a heart attack, or how they reduce inflammation. Therefore, the primary outcome of our study will determine the effect of SGLT2i Empagliflozin on measures of inflammation in patients with T2DM following an AMI, over a 180‐day period with both early (Day 0) and delayed (Day 90) intervention. Specifically, we will look at IL1β mRNA as a measure of NLRP3 inflammasome priming, as well as mRNA expression of several other inflammatory markers. To assess inflammasome activation we will measure caspase‐1 activity, the gold‐standard marker of NLRP3 activity. Additionally, the release of pro‐inflammatory markers IL1β, IL6 and TNFα from MDMs into the supernatant will be evaluated.

There has been recent in vivo evidence that the SGLT2i Empagliflozin prevents cell senescence and circulating SASP markers in murine models of disease.^25^, ^26^ A secondary outcome of this study is to delineate the effects of Empagliflozin on cell senescence and SASP in human patients. To do so, we will measure mRNA levels of p21, p27, BCL2, BAX, IL8 and IL11 in patient macrophages—markers of the cell cycle, programmed cell death and the SASP. In addition, we will also assess circulating levels of SASP biomarkers MMP‐9, OPN, Serpin E1 and MCP‐1 in patient plasma samples.

Having previously linked aberrant Cx43 hemichannel activity to NLRP3 inflammasome activation and cell senescence in in vitro^27^ and in vivo^12^, ^28^ models of kidney disease, we will determine if Empagliflozin inhibits hemichannel‐mediated ATP release to prevent inflammasome activation. Cx43 hemichannel blockers Tonabersat and Peptide 5, each of which work through different mechanisms to prevent the opening of Cx43 hemichannels, will be used to confirm the origin of ATP release.

The strengths of our study include using gold‐standard techniques for measuring various outcome measures, along with novel techniques developed locally on previously established and validated protocols. Whilst many studies examine changes in biomarkers, the response of which can be variable and transitory, the isolation of patient MDMs allows for a more in‐depth study into their behaviour and the underlying immunomodulatory mechanisms of SGLT2i. Limitations include a relatively small patient group number (66) and use of a single SGLT2i. Furthermore, with a strict exclusion criterion, critical patients are not included, meaning we may miss larger beneficial effects that would otherwise be observed in a more critically ill cohort.

## 4 CONCLUSION

Understanding the fundamental processes that protect in vitro and in vivo is critical to explain the clinical benefits seen across the heart and kidneys with SGLT2i therapy. It not only informs on the plausibility of how and why it works to expand our understanding of the disease process and inter‐organ biological pathways but may itself identify future therapeutic targets. Furthermore, assessing how these mechanisms are impacted by the timing of when SGLT2i therapy is initiated will support the notion that early intervention in T2DM at the point of AMI may confer the greatest protection and help inform on how and when to best treat different patient groups. This study will elucidate mechanisms for SGLT2i in protecting patients with T2DM who have recently had an AMI through potential effects on priming/activation of the NLRP3 inflammasome, cell senescence and sustained tissue damage; potentially via aberrant connexin hemichannel activity.

## FUNDING INFORMATION

Project support from EFSD/Boehringer Ingelheim European Research Programme on ‘Multi‐System Challenges in Diabetes’ (CEH, PES and KL). UoL internal PDRA funding support (CLC).

## CONFLICT OF INTEREST STATEMENT

MUS: Honoraria, travel and educational grant from Boehringer Ingelheim.

## Supporting information


Table S1.
