# Supplementary material for: The cardio‐renal‐metabolic role of the nod‐like receptor protein‐3 and senescence‐associated secretory phenotype in early sodium/glucose cotransporter‐2 inhibitor therapy in people with diabetes who have had a myocardial infarction
Source: Diabet Med. 2025 Apr 25;42(7):e70059. doi: 10.1111/dme.70059 (PMC12151815; doi:10.1111/dme.70059)
Supplement: Supplementary file 1 — Table S1. [file DME-42-e70059-s001.docx]

| Gene | Forward | Reverse |
| --- | --- | --- |
| 18S | 5'GTTGGTTTTCGGAACTGAGG3' | 5'GCATCGTTATGGTCGGAAC3' |
| BAX | 5'ATGGACGGGTCCGGGGAG3' | 5'ATCCAGCCCAACAGCCGC3' |
| BCL2 | 5'AACTGTACGGCCCCAGCAT3' | 5'GCCAAACTGAGCAGAGTCTTCAG3 |
| CD80 | 5'GGGAAATGTCGCCTCTCTGAAG3' | 5'ATTGGAGGGTGTTCCTGGGTC3' |
| IL1β | 5'TGGCAGAAGTACCTGAGCTCGC3' | 5'GCCGCCATCCAGAGGGCAGA3' |
| IL6 | 5'CCTGAGAAAGGAGACATGTAACAAGA3' | 5'GGAAGGTTCAGGTTGTTTTCTGC3' |
| IL8 | 5'GAGAGTGATTGAGAGTGGACCAC3' | 5'CACAACCCTCTGCACCCAGTTT3' |
| MCP1 | 5'GCTCGCTCAGCCAGATGCAA3' | 5'TCCTGAACCCACTTCTGCTTG3' |
| P21 | 5'AGGTGGACCTGGAGACTCTCAG3' | 5'AGGTGGACCTGGAGACTCTCAG3' |
| P27 | 5'GGCTTTCAGATTCCCAACTT3' | 5'AGCCTCCCCACTCTCGTCT3' |
| STAT1 | 5'TGTATGCCATCCTCGAGAGC3' | 5'AGACATCCTGCCACCTTGTG3' |
| TNF-α | 5'ATGGGCTACAGGCTTGTCACTC3' | 5'CTCTTCTGCCTGCTGCACTTTG3' |

**Table S1. Forward and reverse human mRNA primer sequences (5'-3') used for RT-qPCR**
